# Editorial: Antimicrobial Photodynamic Therapy: A New Paradigm in the Fight Against Infections

**DOI:** 10.3389/fmed.2021.788888

**Published:** 2021-10-29

**Authors:** Yolanda Gilaberte, Antonio Rezusta, Angeles Juarranz, Michael R. Hamblin

**Affiliations:** ^1^Department of Dermatology, Miguel Servet University Hospital, Institute for Health Research Aragon, Zaragoza, Spain; ^2^Department of Microbiology, Miguel Servet University Hospital, Institute for Health Research Aragon, Zaragoza, Spain; ^3^Department of Biology, University Autonoma of Madrid, Madrid, Spain; ^4^Faculty of Health Science, Laser Research Centre, University of Johannesburg, Johannesburg, South Africa

**Keywords:** photodynamic therapy, infections, bacteria, photoinactivation, antimicrobial, SARS.Cov-19

According to a 2019 report from the Center for Disease Control and Prevention (CDC), more than 2.8 million antibiotic-resistant infections occur in the U.S. each year, and more than 35,000 people die as a result. This special issue is focused on Antimicrobial Photodynamic Therapy (PDT), which is a new strategy to fight against infections. PDT is mostly used in the treatment of cancer, and non-melanoma skin cancer is the most widely recognized indication. A photosensitizer that is activated by visible light in the presence of oxygen can generate reactive oxygen species resulting in the death of the microorganisms, without damaging the surrounding tissue. This innovative way of destroying microbial pathogens has many advantages compared with the conventional antimicrobials and antibiotics used so far. It has a broad spectrum of action, being able to kill or inactivate bacteria, fungi, viruses and protozoa. The killing action of PDT against microbes is immediate, while most antibiotic drugs take hours or days to work. PDT can photoinactivate microorganisms independent of their pattern of antimicrobial resistance, and has a low likelihood of selecting drug-resistant strains due to its multitarget mechanism of action. These advantages have made PDT an appealing option worth exploring in the future fight against infections.

The present special issue is a collection of original and review articles covering different aspects of aPDT, from the use of nanoplatforms and polymers to the treatment of wounds with a combination of classical antimicrobials plus methylene blue. We hope that these articles will serve to increase the evidence in support of aPDT, and to improve the clinical management of patients with infections.

## Research Highlights

*Stimuli-responsive nanoplatform assisted photodynamic therapy against bacterial infections (Zhou et al.)*. The review by Zhou et al. is focused on the use of nanoplataforms to improve the activity of PDT against bacterial infections. The abuse of antibiotics and the emergence of resistant microbes are causing a great number of deaths in clinical settings. They point out the importance of PDT to treat these infections and to reduce bacterial persistence in biological tissues. The microenvironment in bacterial infected tissues is unique, and allows the design of responsive nanoplatforms, which can provide a promising strategy for drug delivery. Drug delivery nanoplataforms and, in particular stimulus-responsive nanoplatforms (such as, pH, enzymes, redox gradients, magnetic, and electric fields) can enhance the efficacy of conventional antibacterial PDT, by increasing the selectivity and specificity of photosensitizers as a promising way to combat bacterial infections.

*Comparison of antibacterial activity and wound healing in a superficial abrasion mouse model of S. aureus skin infection using photodynamic therapy based on methylene blue or mupirocin or both (Pérez et al.)*. Pérez et al. show in this manuscript that methylene blue (MB) is a good photosensitizer for PDT in superficial wounds in mice infected by *S. aureus*. After comparing 1 session of MB-PDT vs. topical mupirocin, or the combination of both, MB-PDT followed by daily mupirocin, they found that whereas MB-PDT provided the quickest and the most cosmetic wound healing, mupirocin showed the highest logarithmic reduction of *S. aureus* CFUs. The combination of both therapies improved the antimicrobial activity of MB-PDT alone, but without showing a synergistic effect or improving the wound healing results provided by MB-PDT alone.

*Toward universal photodynamic coatings for infection control (Ghareeb et al.* The COVID-19 pandemic and hospital-acquired infections have increased the interest in self-disinfecting materials for infection control. The antimicrobial efficacy of the coated materials was evaluated against methicillin-susceptible *S. aureus* ATCC-29213 and human coronavirus strain HCoV-229E. The study showed that a spray coated photosensitizer could be applied to a wool/polyester/polyamide upholstery fabric combination. The study suggest that photodynamic spray coatings may be a simple but effective tool for reducing the transmission of pathogens in healthcare settings. The longevity of the activity of SbQ-PVA/PS, after exposure to environmental light, suggests that such coatings could remain effective against multiple pathogen exposures during weeks.

*Factors determining the susceptibility of bacterial to antibacterial photodynamic inactivation (Rapacka-Zdończyk et al.)*. Rapacka-Zdończyk et al. recognize that despite aPDT theoretically being effective against all types of bacteria, some of them are more susceptible than others. They discuss the potential problem that aPDI or antimicrobial blue light can cause damage to DNA, which could theoretically lead to resistance to the treatment. The authors also discuss the different effects on the bacteria if the photosensitizer is either in solution or else is attached on solid material which can or cannot enter the bacterial cell, respectively. In the latter case, the photosensitizer can destroy external cell structures, including the cytoplasmic membrane in Gram-positive bacteria, and the cytoplasmic and outer membrane in Gram-negative bacteria, which lead to the death of the microbial cells.

*Broad-spectrum photo—antimicrobial polymers based on cationic polystyrene and Rose Bengal (Gavara et al.)*. Gavara et al. suggests that a new strategy to fight bacteria and fungi is necessary in view of the problem of nosocomial infections combined with the growing threat of increased antimicrobial resistance. The authors studied the combination of Rose Bengal (RB) with two polymers (macroporous resin Amberlite® IRA 900 or gel-type resin IRA 400). The most interesting finding was that these preparations were able to reduce the population of both Gram-positive and Gram-negative bacteria after photoactivation, although, for *C. albicans*, to a moderate degree. RB had been considered ineffective for the inactivation of Gram-negative bacteria, but when it was combined with commercial supports such as cationic exchange resins, the resultant materials could be effective against all bacterial pathogens. The study opens the possibility to use these materials for the development of coatings for self-disinfecting surfaces.

*Spatial distribution of a porphyrin-based photosensitizer reveals the mechanism of photodynamic inactivation of Candida albicans (Voit et al.)*. The article published by Voit et al. demonstrates the efficacy of the porphyrin [5,10,15, 20-tetrakis(1-methylpyridinium-4-yl)-porphyrin tetra p-toluenesulfonate] (TMPyP) in combination with light for the treatment of *Candida albicans*. Although TMPyP has been identified as an effective PS for photokilling of *C. albicans*, the antifungal mechanism of action that led to inactivation of the yeast is not well-understood. In this sense, the authors evaluated the localization kinetics of TMPyP (before and after irradiation) with blue light, showing that the red fluorescence of TMPyP appeared in the fungal cell envelope, and after irradiation the fungal cell wall was damaged, and TMPyP relocalized into the cytosol. The authors suggest that the accumulation of the photosensitizer in the cell wall of yeast could avoid resistance after the treatment, and increase the PDT efficacy against *C. albicans* and probably other fungal infections.

## Author Contributions

All authors listed have made a substantial, direct and intellectual contribution to the work, and approved it for publication.

## Conflict of Interest

The authors declare that the research was conducted in the absence of any commercial or financial relationships that could be construed as a potential conflict of interest.

## Publisher's Note

All claims expressed in this article are solely those of the authors and do not necessarily represent those of their affiliated organizations, or those of the publisher, the editors and the reviewers. Any product that may be evaluated in this article, or claim that may be made by its manufacturer, is not guaranteed or endorsed by the publisher.

